# Proposed Definitions of T Cell-Mediated Rejection and Tubulointerstitial Inflammation as Clinical Trial Endpoints in Kidney Transplantation

**DOI:** 10.3389/ti.2022.10135

**Published:** 2022-05-20

**Authors:** Daniel Seron, Marion Rabant, Jan Ulrich Becker, Candice Roufosse, Maria Irene Bellini, Georg A. Böhmig, Klemens Budde, Fritz Diekmann, Denis Glotz, Luuk Hilbrands, Alexandre Loupy, Rainer Oberbauer, Liset Pengel, Stefan Schneeberger, Maarten Naesens

**Affiliations:** ^1^ Department of Nephrology and Kidney Transplantation, Vall d’Hebrón University Hospital, Barcelona, Spain; ^2^ Department of Pathology, Hôpital Necker–Enfants Malades, Paris, France; ^3^ Institute of Pathology, University Hospital Cologne, Cologne, Germany; ^4^ Centre for Inflammatory Disease, Department of Immunology and Inflammation, Imperial College London, London, United Kingdom; ^5^ Department of Surgical Sciences, Sapienza University of Rome, Rome, Italy; ^6^ Division of Nephrology and Dialysis, Department of Internal Medicine, Medical University of Vienna, Vienna, Austria; ^7^ Department of Nephrology and Medical Intensive Care, Charité Universitätsmedizin Berlin, Berlin, Germany; ^8^ Department of Nephrology and Kidney Transplantation, Hospital Clinic Barcelona, Barcelona, Spain; ^9^ Paris Translational Research Center for Organ Transplantation, Hôpital Saint Louis, Paris, France; ^10^ Department of Nephrology, Radboud University Medical Center, Nijmegen, Netherlands; ^11^ Paris Translational Research Center for Organ Transplantation, Hôpital Necker, Paris, France; ^12^ Department of Nephrology and Dialysis, Medical University of Vienna, Vienna, Austria; ^13^ Centre for Evidence in Transplantation, Nuffield Department of Surgical Sciences, University of Oxford, Oxford, United Kingdom; ^14^ Department of General, Transplant and Thoracic Surgery, Medical University of Innsbruck, Innsbruck, Austria; ^15^ Department of Microbiology, Immunology and Transplantation, KU Leuven, Leuven, Belgium

**Keywords:** kidney transplantation, outcomes, EMA guideline, T cell-mediated rejection, borderline changes

## Abstract

The diagnosis of acute T cell-mediated rejection (aTCMR) after kidney transplantation has considerable relevance for research purposes. Its definition is primarily based on tubulointerstitial inflammation and has changed little over time; aTCMR is therefore a suitable parameter for longitudinal data comparisons. In addition, because aTCMR is managed with antirejection therapies that carry additional risks, anxieties, and costs, it is a clinically meaningful endpoint for studies. This paper reviews the history and classifications of TCMR and characterizes its potential role in clinical trials: a role that largely depends on the nature of the biopsy taken (indication vs protocol), the level of inflammation observed (e.g., borderline changes vs full TCMR), concomitant chronic lesions (chronic active TCMR), and the therapeutic intervention planned. There is ongoing variability—and ambiguity—in clinical monitoring and management of TCMR. More research, to investigate the clinical relevance of borderline changes (especially in protocol biopsies) and effective therapeutic strategies that improve graft survival rates with minimal patient morbidity, is urgently required. The present paper was developed from documentation produced by the European Society for Organ Transplantation (ESOT) as part of a Broad Scientific Advice request that ESOT submitted to the European Medicines Agency for discussion in 2020. This paper proposes to move toward refined definitions of aTCMR and borderline changes to be included as primary endpoints in clinical trials of kidney transplantation.

## Acute T Cell-Mediated Rejection Endpoints: The History

Health authorities have long accepted biopsy-proven acute rejection (BPAR) as a primary efficacy variable in clinical trials for the prevention and treatment of transplant rejection (1): in epidemiological studies performed during the 1990s (2, 3), BPAR was associated with poor long-term outcomes. There is a general belief that BPAR is considered to reflect acute T cell-mediated rejection (aTCMR), likely in part related to the fact that between 1991 and 2001 the recognition of antibody-mediated rejection (AMR) was limited to its hyperacute/accelerated forms and overshadowed by grading of aTCMR in the Banff Classification for Allograft Pathology (4). This belief extends to the fact that many pivotal studies of immunosuppressant therapy have utilized the term “BPAR” to describe what was often more specifically aTCMR, identified on indication biopsies (discussed below). Indeed, the opinion that BPAR and aTCMR are interchangeable terms remains largely speculative; evidence indicates that they are not equal, if only because the definition of BPAR does not discriminate between the different subtypes of rejection that have been identified (see Becker et al. (5), this special issue).

However, as AMR was only introduced into the Banff Classification later (4), and as the specific definition of aTCMR has not changed markedly since 1997, with some caveats the aTCMR diagnosis can likely be used for between-study comparisons over time. The fact that aTCMR is managed with antirejection therapies that cause risk, anxiety, and cost continues to make aTCMR a clinically relevant endpoint for research purposes. TCMR was found to be an important cause for graft failure in a recent retrospective study (6). Of note, interobserver variability in the diagnosis of aTCMR is high (7), which warrants caution in the interpretation of single-center data without central pathological review.

In terms of drug development studies in kidney transplantation, European Medicines Agency and US Food & Drug Administration approvals of mycophenolate mofetil, daclizumab, tacrolimus, basiliximab, and sirolimus were based primarily on superiority findings, with BPAR (more specifically, rates of aTCMR in indication biopsies) included as the primary efficacy variable or part of a composite measure, with graft failure and patient death (8–11). In the Symphony study (12), 1,645 kidney transplant recipients were randomized to combination therapy involving mycophenolate mofetil and corticosteroids, with or without cyclosporine, daclizumab induction, tacrolimus, or sirolimus; kidney function (evaluated by estimated [e] glomerular filtration rate [GFR] at 1 year post transplantation) was the primary efficacy variable and BPAR was the secondary efficacy variable. Kidney function and graft survival rates were better in tacrolimus-treated patients compared with others: the BPAR rate (of unspecified subtype; presumably mostly aTCMR) was lowest in those receiving low-dose tacrolimus (12%) compared with standard-dose cyclosporine (26%), low-dose cyclosporine (24%), or low-dose sirolimus (37%) (12).

The Symphony trial therefore defined a new standard of care in kidney transplantation that was widely employed thereafter because of its efficacy in preserving function and preventing rejection. Subsequent studies also reported similarly low rates of BPAR for innovative combination regimens (13–17). Collectively, this research showed that the incidence of BPAR in indication biopsies could be modulated by immunosuppressive therapy and has decreased considerably over time, leading to improvements in post-transplantation treatment and understanding of acute rejection (18).

## Acute TCMR in Indication Biopsies in Superiority or Non-Inferiority Studies

Now that the incidence of aTCMR is consistently reported at ∼10% during the first year following kidney transplantation (19–21), it is important to reconsider its utility as a primary efficacy variable. The low prevalence of aTCMR with current immunosuppressive regimens, and the less consistent association of aTCMR with outcome (22–26), indicate a limited need for superiority trials that aim to further reduce rates of aTCMR. Any benefits gained from such trials would be outweighed by the considerable drawbacks associated with powerful regimens that risk over-immunosuppression and create safety or tolerability issues for many patients. Nevertheless, including biopsy-proven aTCMR as a primary efficacy variable in non-inferiority trials remains highly relevant, since aTCMR in an indication biopsy leads to heightened therapeutic interventions, treatment burden, morbidity, and cost. Treatment-resistant TCMR may also lead to graft loss, or in less severe cases to nephron loss, with detrimental long-term consequences for graft function.

Definition and presentation of the rejection subtype, and its association with outcome, are important considerations for discussions exploring the value of aTCMR as a primary efficacy variable in clinical trials. For example, AMR was not clearly defined until 2001 (4): it is likely that some patients considered to have BPAR in the 1990s might have experienced an unrecognized episode of AMR or mixed AMR–aTCMR. Consequently, the relationship between BPAR (i.e., aTCMR) and outcome may have been overemphasized in the past.

In addition to the rejection subtype, one can also reflect on how characteristics of donors and recipients, and the incidence of rejection, have changed in a time-dependent manner with emerging evidence and improvements in practice. Studies evaluating the relationship between aTCMR and graft survival have therefore yielded seemingly contradictory results (22–25): they indicate that further reductions of the incidence of aTCMR will not directly translate into better rates of long-term graft survival, and also suggest that higher incidences of aTCMR do not correlate with incidences of graft failure.

For example, in an epidemiological study that distinguished between aTCMR and AMR, aTCMR diagnosed by indication biopsies was not associated with decreased graft survival rates (22). In the Tricontinental Mycophenolate Mofetil Renal Transplantation Study, outcome evaluation at 3 years (i.e. 3-year graft survival rate) did not show any benefit for cyclosporine plus mycophenolate mofetil over cyclosporine plus azathioprine, despite a significant reduction in the incidence of rejection during the first year. However, this study was not adequately powered to detect a difference in 3-year graft survival rates (23). Of note, patients included in the Tricontinental study in Australia were followed for 15 years; again, no long-term benefit of mycophenolate mofetil was observed (24).

Conversely, in a 5-year follow-up of a study comparing steroid continuation or withdrawal in a tacrolimus plus mycophenolate mofetil-based regimen, the acute rejection rate increased after steroid withdrawal and was associated with decreased survival (25). Analysis of the Australian and New Zealand Dialysis and Transplant Registry (13,614 recipients) showed that aTCMR was associated with allograft survival and death with a functioning graft (specifically, death due to cardiovascular disease or cancer) (26).

Similarly, in the belatacept trial (27), more-intensive and less-intensive belatacept regimens were compared with a cyclosporine-based regimen, with BPAR, graft loss, and recipient death as the composite primary endpoint that was used to demonstrate non-inferiority, and GFR as the endpoint to show superiority. Despite higher incidence of BPAR during the first year (22% in the more-intensive belatacept group, 17% in the less-intensive belatacept group, 7% in the cyclosporine group), kidney function and long-term allograft survival were superior in patients receiving belatacept. However, when belatacept- or cyclosporine-treated recipients with or without rejection were compared, GFR was significantly lower in those who experienced an episode of acute rejection, suggesting nephron loss in patients experiencing BPAR (or aTCMR). This supports the prognostic meaning of rejection, even in patients on belatacept. The proportion of patients who developed *de novo* (*dn*) donor-specific antibodies (DSA) at 7 years was decreased in belatacept-treated patients compared with cyclosporine-treated patients (28), illustrating that most BPAR cases were aTCMR, and that a higher rate of aTCMR did not translate into a worse outcome in this trial. The poorer kidney function in cyclosporine-compared with belatacept-treated patients could be explained by nephrotoxicity, not rejection, although rejection still affected graft function within the belatacept arm. It is unclear whether lower *dn*DSA, despite higher aTCMR, could be explained by a specific effect of belatacept, or better adherence to this treatment compared with cyclosporine.

Mixed results on the association between aTCMR and outcome were corroborated by indication-biopsy findings reported for 256 kidney transplant recipients with aTCMR, treated with steroids (29). Overall graft survival rates were 85% after 5 years and 69% after 10 years. Best predictors of allograft loss were GFR, presence of inflammation in areas of interstitial fibrosis and tubular atrophy (i-IFTA) on 3-month protocol biopsies, and presence of anti-HLA (human leukocyte antigen) DSAs at 3 months. This suggests that transition from aTCMR to chronic active (ca)TCMR or response to aTCMR treatment constitute hallmarks of poor long-term outcome; it also illustrates that not all aTCMR episodes are equal, at least in terms of their treatment response. For example, patients with aTCMR (on indication biopsy) and a GFR >44 ml/min, no or mild i-IFTA, and no anti-HLA-DSA had a 74% graft survival rate at 10 years, whereas those with aTCMR and i-IFTA grade 2 or 3 had a 55% graft survival rate at this time point (29). It is obvious that aTCMR may cause injury to the nephron, which might result in subsequent nephron loss, as evidenced by the fact that aTCMR contributed to graft loss in ∼34% of failures (6). Older data also suggested that aTCMR grade II [with intimal arteritis) conferred less responsiveness to steroid therapy and a poorer prognosis for graft survival than grade 1 aTCMR (30, 31)]. Notwithstanding these data, more research is needed to better define the aTCMR phenotypes that confer increased risk of worse outcome, which is potentially of importance for the choice of the primary efficacy variable in future clinical trials.

## Borderline Changes in Indication Biopsies

As the presence of aTCMR in indication biopsies is perhaps less important than it was in the early years of kidney transplantation, the relevance of borderline changes in such biopsies might be even more trivial, given that these represent less severe inflammation scores than aTCMR. Nevertheless, sampling errors and low reproducibility of Banff Lesion Scores could lead to arbitrary classifications, and less strict distinctions between aTCMR and borderline changes that do not reach the aTCMR threshold. This is corroborated by molecular analysis of biopsies showing borderline changes at the time of graft dysfunction, which illustrates that such changes represent a molecularly heterogeneous group: some do not resemble rejection, whereas others resemble aTCMR (32).

Indication biopsies are undertaken when there are clinical signs of deteriorating kidney function. Although borderline changes detected on indication biopsies are less likely to be associated with graft failure than aTCMR, 50–80% of cases of borderline changes detected in indication biopsies receive antirejection treatment with high-dose corticosteroids (33–35). Consequently, borderline changes can be clinically relevant even in the absence of more severe lesions, because of the impact of any decision to initiate antirejection therapy (36).

Despite this clinical relevance, the association between borderline changes in indication biopsies and graft outcome has not been widely studied in the current context of transplantation medicine; the limited research findings are mixed. A retrospective analysis illustrated that graft survival rates were significantly better in patients with borderline changes than in those with aTCMR, but significantly worse than in the control group, despite antirejection therapy, similar clinical characteristics, and similar graft dysfunction at time of biopsy (37). More recently, comparison of patients with different lesion scores for borderline rejection showed that the occurrence of death-censored graft failure or doubling of serum creatinine concentration post biopsy at 5 years was 5% for those scoring t1i0 but reached 14% for those scoring ≥t1i1. These endpoints also occurred in 5% of recipients with no rejection and 21% of those with TCMR. Patients with biopsy lesion scores of t1i0 therefore had a prognosis similar to that of non-rejectors (adjusted hazard ratio [HR] 0.6; 95% confidence interval [CI] 0.1–2.2), and better than that of patients with lesions scoring ≥t1i1 (adjusted HR 3.8; 95% CI 1.3–11.5) (4).

In a study of 803 renal transplantations, Wiebe et al. found an independent correlation between HLA-DR/DQ molecular mismatch, presence of borderline changes (diagnosed according to the Banff 1997 definition), and severity and frequency of rejection episodes. These investigators suggested that borderline changes could be part of a spectrum of alloimmune-mediated inflammation, not simply a response to injury (38).

The place of borderline changes as an endpoint in clinical trials is therefore not entirely clear, but there is evidence of its clinical relevance as far as observed in indication biopsies. Many registration studies for immunosuppressant therapies in kidney transplantation that utilized BPAR as the endpoint did not specify either the grade of rejection or the inclusion of borderline changes (8, 39–45). In registration studies for basiliximab (46,47) and belatacept (48), the definition of BPAR excluded borderline changes; only grades I or II aTCMR were considered in the BPAR definition (49). Only the ZEUS trial included borderline changes in its BPAR definition (49).

In Wu et al.’s retrospective analysis, borderline changes were treated with antirejection drugs, leading to complete reversibility in 57%, partial reversibility in 39%, and no reversibility in only 4% of cases (vs. 15% and 21% no reversibility for TCMR grades I and II, respectively) (37). Similarly, an earlier and smaller retrospective study (25) reported a high likelihood of complete response with antirejection treatment for borderline changes. Finally, in another retrospective study, outcome after determination of borderline changes (by serum creatinine and/or subsequent histology) showed that untreated changes were non-progressive in 72% of cases (50), although some biopsies were performed per protocol and thus not considered indication biopsies. This study suggested that conservative management of borderline changes in indication biopsies, at least in the short term, might be more appropriate than routine treatment as indicated for acute rejection.

As these were retrospective studies with mixed results, no conclusions can be drawn on the necessity or timing of any treatment for borderline changes in indication biopsies. The decision depends on center practice and clinician’s judgment. However, the participants at the Banff 2019 meeting agreed that any findings below the i1t1 threshold would not be considered borderline changes as they are not associated with impaired graft outcome. In addition, as antirejection therapy is associated with treatment burden, comorbidity, anxiety, and heightened cost, a diagnosis of borderline changes that leads to therapeutic intervention represents a clinically impactful event. In a clinical trial setting, this could be a relevant marker for evaluating non-inferiority, despite having a limited association with graft failure and higher likelihood of reversibility on treatment, compared with aTCMR.

## Borderline Changes or TCMR in Protocol Biopsies

The potential utility of protocol biopsy histology as a primary efficacy variable has been evaluated in clinical trials of interventions that aim to prevent subclinical inflammation during the first year following kidney transplantation. Here, we consider the association between subclinical inflammation and outcome in patients not treated or treated for this condition, and the effect of basal immunosuppression on the incidence of subclinical inflammation. Since there is no international consensus on the definition of protocol biopsies, and the definitions used are not always explicitly stated in papers, there is some heterogeneity in the literature. Some centers define protocol biopsies according to the prescheduled nature of the biopsy; others take graft functional characteristics into account. For future clinical trials, the term “protocol biopsy” should be defined precisely, in terms of allowed change in serum creatinine or proteinuria, to improve interpretability of the results. International standardization of the definition of protocol biopsy would be highly welcomed.

Subclinical rejection (and/or borderline changes), identified in protocol biopsies during the first year post transplantation, are associated with progression of IFTA and increased serum creatinine levels (51, 52), impaired glomerular adaptation (53, 54), *dn*DSA appearance (52–56), and decreased graft survival (57).

Presence of borderline changes alone is associated with persistent inflammation in serial protocol biopsies, IFTA progression (58), *dn*DSA appearance, and decreased graft survival 51]. Nankivell et al. compared 146 patients with borderline changes (92 subclinical and 54 clinical episodes) versus 826 normal controls and 55 aTCMR patients. Subclinical borderline changes improved on subsequent protocol biopsies in 72% of cases but persisted in 19% and worsened in 9%. Untreated subclinical borderline changes resolved in 62% of cases, persisted in 27%, and worsened in 12%. Overall, presence of borderline changes remained an independent predictor of graft failure when adjusted for multiple immunological risk factors, time since transplant, and biopsy indication (51). However, the retrospective and associative nature of these data is a clear limitation of these studies, and bias introduced by attending physicians in treatment decision-making means the findings should be interpreted cautiously.

In a large study of 1-year protocol biopsies conducted in patients transplanted between 2000 and 2010, 73% of patients did not show rejection (with borderline changes counted as no rejection), 13% showed aTCMR (i ≥ 2 and t ≥ 2), and 14% showed AMR; graft survival rate decreased significantly in patients with AMR (59). However, protocol biopsies indicate that graft survival rates at 1 year were no different in patients with aTCMR than in those without rejection. This illustrates that evaluation of the rejection subtype is key, and that subclinical aTCMR and subclinical AMR should not be considered a single entity. Notably, all patients with subclinical aTCMR received steroid boluses according to routine practice (59). Favorable outcomes in patients with subclinical aTCMR could be explained by treatment effects, but no conclusions could be drawn about the impact of untreated subclinical aTCMR on graft outcome. Since borderline changes were not analyzed separately in this study, no conclusions can be drawn about the influence of subclinical borderline changes on outcome.

Although a randomized study showed that cyclosporine plus mycophenolate and steroids was associated with a higher risk for subclinical rejection (borderline changes and aTCMR) than tacrolimus, subclinical rejection was determined retrospectively, and was therefore untreated (60). Nevertheless, despite lack of treatment, this study showed that subclinical rejection did not lead to differences in graft functional evolution or fibrosis (60). Importantly, subclinical inflammation evaluated in non-fibrosed areas of protocol biopsies already displaying IFTA is more closely associated with poor graft survival than inflammation in otherwise normal biopsies (54, 58, 61–64). Finally, a randomized multicenter study from Canada indicated that subclinical TCMR or borderline changes occurred in 30–50% of patients at 6 months following transplant, depending on the level of tacrolimus dosing and the use of angiotensin-converting enzyme inhibitors/angiotensin II receptor 1 blockers (ACEi/ARBs), compared with other anti-hypertensive regimens. Patients with the lowest subclinical rejection risk (low-dose tacrolimus plus ACEi/ARBs) had reduced risk of progression of IFTA (65). It should be noted that this association did not necessarily indicate a causal relation between reduced risk of subclinical rejection and reduced IFTA progression.

Since the 2017 Banff Classification, caTCMR has been defined for the tubulointerstitial compartment, in part based on the grade of inflammation in atrophic areas (Banff i-IFTA score) (66). This histological phenotype in protocol biopsy studies is often preceded by interstitial inflammation in non-IFTA areas, coexists with interstitial inflammation in healthy areas, and constitutes a risk factor for progression of fibrosis and shortened graft survival (67, 68). Analysis of 1,500 1-year protocol biopsies by the Paris transplant group revealed that of the 893 biopsies scored as IFTA ≥1, 518 had no i-IFTA, 181 had an i-IFTA 1, and 194 had moderate to severe i-IFTA (2 or 3). Moderate to severe i-IFTA was associated with a decreased long-term graft survival rate and i-IFTA was superior to i, ti, and t scores for predicting allograft failure in patients with fibrosis at 1 year. In the Paris study, determinants of i-IFTA at 1 year were previous episodes of TCMR or BK virus nephropathy, as well as under-immunosuppression (67). As this indicates that both over- (e.g., polyomavirus nephropathy) and under-immunosuppression cause the same phenotype of chronic tubulointerstitial injury, it is difficult to establish the causes of that chronic injury.

Nankivell et al. reviewed i-IFTA in 2,481 biopsies from 362 patients, which were mainly protocol biopsies (mean number of seven biopsies per patient). Sequential histology demonstrated that interstitial inflammation occurred before the appearance of i-IFTA and chronic fibrosis. The 1-year i-IFTA intensity correlated with the number of prior TCMR episodes (68). In this study, i-IFTA was also associated with a worse graft survival rate and worse kidney function. Of note, however, although these data illustrated associations between caTCMR and graft failure, they were from retrospective studies. This limits the interpretability of results regarding whether it is necessary to treat, or not treat, such episodes of rejection. More recent data even indicate that most i-IFTA lesions are not preceded by rejection, and that even when they are, this rejection could be either TCMR or AMR (69). Finally, grade II caTCMR (chronic allograft arteriopathy, arterial intimal fibrosis with mononuclear cell inflammation in fibrosis, and formation of neointima) is even less well defined than other rejection subtypes, and may also be a manifestation of caAMR, cAMR, or mixed AMR/TCMR. Taken together, it is anticipated that further refinement of the diagnostic criteria of caTCMR will be important, both for clinical decision-making and before such criteria could be considered for clinical trial endpoints (70).

## Incomplete Inflammatory Phenotypes

Since tacrolimus and mycophenolate were introduced, the prevalence of tubulointerstitial inflammation in the first 2 years (subclinical aTCMR and borderline changes) has fallen from >50% to ∼10% of transplant recipients (71). In addition, severity of inflammation has decreased to the point that subclinical aTCMR in protocol biopsies constitutes an uncommon diagnosis (63). Transplant biopsies with inflammation typically show changes or incomplete inflammatory phenotypes that are below the threshold for defining borderline changes (61, 66), raising the question whether such findings have any association with graft survival.

In an investigation of the clinical and pathological significance of borderline changes, lesions under i1 were excluded; when the significance of isolated tubulitis (*i* = 0, t ≥ 1) on outcome was evaluated, no relationship was found between this lesion type and kidney allograft survival rate (51). Consequently, it was suggested not to include isolated tubulitis in the borderline category. This decision was agreed at Banff 2019, and included in the Banff criteria accordingly (72).

In another study including 200 of 275 patients with a 3-month protocol biopsy who did not meet the Banff criteria for TCMR grade IA, patients were classified as either no inflammation (i0t0) or inflammation (i + t > 1). Compared with transplant recipients without inflammation, those with inflammation showed higher chronic scores at 1 year, higher serum creatinine levels at 2 years, and higher incidence of *dn*DSA (73). In a further study, these authors illustrated that although these incomplete phenotypes of rejection were associated with increased risk of subsequent aTCMR, there was no association with worse graft survival (74). Notably, the lack of unified treatment protocol and small sample size hamper the interpretation of these results, and further research is warranted.

## Impact of Treating Subclinical Inflammation

From the findings discussed above, we can conclude that the different histological phenotypes of subclinical inflammation in protocol biopsies − aTCMR, borderline changes, caTCMR, and interstitial inflammation without tubulitis—have been associated with decreased graft survival rates in retrospective and observational cohort studies. Whether such subclinical inflammation should be recognized as pathology requiring treatment merits further discussion. A pioneering evaluation of steroid bolus treatment for kidney transplant recipients with subclinical aTCMR and borderline changes (t1/2/3 + i0/1 or t1 + i2/3) randomized participants to receive either biopsy at 1, 2, and 3 months with steroid treatment of subclinical inflammation, or no biopsy and no steroid treatment at 1, 2, and 3 months (75). Both groups had a 6-month protocol biopsy, received the then-standard of care immunosuppression with cyclosporine and azathioprine, and were followed for 2 years. Patients in the biopsy group had fewer cases of fibrosis at the 6-month protocol biopsy and better kidney function at 2 years, suggesting that treatment of subclinical inflammation preserves kidney structure and function. In this study, subclinical inflammation was present in ∼50% of patients. These older data, with an outdated immunosuppressive regimen, suggested that detection of subclinical inflammation permits early, successful treatment and would be useful to include in future interventional trials. Another important weakness of this study is that it does not address the threshold of inflammation above or below which treatment improved (or failed to improve) kidney function at 2 years.

Subsequently, Kurtkoti et al. (76) designed a prospective randomized trial to evaluate whether treatment of rejection in protocol biopsies at 1 and 3 months preserved 1-year kidney function; participants also received cyclosporine and azathioprine. Rates of subclinical aTCMR and borderline changes were ∼15% for each diagnosis at 1 and 3 months. The group of patients in whom treatment was adapted in response to protocol biopsy findings had better kidney function at 1 year, again suggesting that treating subclinical inflammation may improve outcome, although the effect on long-term graft failure was not studied.

As studies with older immunosuppressive regimens suggested treating subclinical inflammation with steroids (75, 76), Rush et al. performed a trial following the same design, but with a tacrolimus and mycophenolate immunosuppressive regimen (77). Although treating subclinical rejection (and rarely borderline changes) at 1, 2, or 3 months had no effect on interstitial fibrosis at 6 months or on kidney function at 12 and 24 months, the prevalence of subclinical inflammation at 1, 2, and 3 months was <10%, which was lower than expected. There is no mention of borderline changes that were not treated. Despite the low numbers of rejections, and in contrast to the hypothesis, the treatment arm tended to have higher chronic scores than the control arm, suggesting that treating subclinical rejection does not halt progressive chronic injury in patients receiving baseline immunosuppression with tacrolimus and mycophenolate (77). From this study, nothing can be implied about the impact of borderline changes in protocol biopsies early after transplantation.

Utility of 3- and 6-month protocol biopsies to predict graft survival was analyzed retrospectively in a pediatric population. Immunosuppression was increased (sometimes with steroid boluses) in patients with subclinical rejection. However, in those with borderline changes, treatment was selected by the attending physician; one-third of borderline episodes were not treated. The probability of reaching the composite outcome variable (i.e., an episode of clinical BPAR or graft failure in the next 5 years) was significantly higher in patients with untreated borderline changes than in treated patients (78). The retrospective nature of this study again warrants cautious interpretation of the data, especially regarding the effect of therapy, which was confounded by the decision of the attending physician.

Although it has been hypothesized that i-IFTA at 1 year is associated with under-immunosuppression (66), it is unclear whether increasing the immunosuppressive regimen or giving steroid-based antirejection therapy prevents or treats this condition. Importantly, immunosuppressive treatment may cause over-immunosuppression, which can create the same histological picture, through events such as the development of polyomavirus nephropathy.

In 1-year protocol biopsy studies performed in patients receiving current standard-of-care immunosuppression, the prevalence rates for subclinical inflammation are: ∼3% for aTCMR (very low), 10%–15% for borderline changes, 15%–20% for incomplete phenotypes and 10% for caTCMR (51, 79, 80). The low frequency of aTCMR should be considered in studies that aim to reduce the incidence of TCMR further. Although treatment of aTCMR or borderline changes in protocol biopsies were suggested to be associated with improved outcome in an earlier era, this cannot be confirmed in studies using current immunosuppressive regimens. Previous heterogeneity in definitions of histological thresholds for the diagnosis of borderline changes (81) and interobserver variability also create additional problems for interstudy comparison.

Because of this heterogeneity in the literature, large variability in clinical practice remains (33–36). Some transplantation centers perform protocol biopsies and routinely treat subclinical aTCMR or borderline changes by increasing immunosuppression or using steroid boluses. Other centers would not treat borderline changes found on protocol biopsies unless there was additional evidence of rejection. A third group of centers do not perform protocol biopsies at all, and therefore never detect or treat subclinical changes. No data are available on European heterogeneity in this respect. Although i1t1 borderline changes in protocol biopsies are associated with a significant risk of subsequent TCMR, it remains unclear whether routinely treating caTCMR or incomplete phenotypes would improve graft outcome, as there is very limited literature on this phenotype.

## Conclusions

This paper reviews the history and classifications of TCMR and characterizes its potential role in clinical trials. ESOT has come to the following recommendations:

• BPAR and aTCMR are not equivalent: although many pivotal studies utilize BPAR to describe findings that could be aTCMR, some may be AMR or chronic rejection subtypes.

 ○ However, the specific definition of aTCMR has changed little over time, and can still broadly be used for longitudinal between-study comparisons.

• Acute TCMR (IA, IB, IIA, IIB, III) diagnosed in indication biopsies should remain included as a primary (non-inferiority) endpoint in clinical trials of kidney transplantation.
• Acute TCMR (IA, IB, IIA, IIB, III) diagnosed in protocol biopsies may be considered as a primary efficacy variable in clinical trials of kidney transplantation.
• Borderline changes (Banff Category 3 in the 2019 definition, restricted to Banff t ≥ 1 + i ≥ 1) diagnosed in indication biopsies following kidney transplantation are usually treated with antirejection therapy, and could be included as a primary (non-inferiority) efficacy variable in clinical trials for kidney transplantation.

 ○ Such borderline changes in indication biopsies are clinically relevant because of the impact of antirejection (immunosuppressive) therapy, which is required in a substantial number of cases diagnosed on indication biopsies.

• Diagnosis of at least one clinical episode of aTCMR or borderline changes (Banff Category 3 in the 2019 definition, restricted to Banff t ≥ 1 + i ≥ 1) in an indication biopsy, or aTCMR in a protocol biopsy, could be proposed as part of a composite primary efficacy endpoint in clinical trials aimed at preventing kidney transplant rejection.
• Few centers treat borderline changes identified in protocol biopsies with antirejection therapy; such changes are less clearly associated with outcome and should not be considered as primary efficacy measures in clinical trials for kidney transplantation.
• Awaiting further evidence, caTCMR (IA, IB, II) and tubulointerstitial inflammation below the Banff threshold for borderline changes should not be considered as measures of efficacy in clinical trials.

### Scientific Advice From the Committee for Medicinal Products for Human Use (CHMP) of the European Medicines Agency (EMA) with regard to These Conclusions

• The CHMP agreed with ESOT that the histological type of rejection (aTCMR, borderline changes, AMR) is a useful specification and that this detailing might be very informative in profiling efficacy of immunosuppression for kidney transplantation.
• The CHMP acknowledged the proposed clinically meaningful definition of borderline changes (to borderline suspicious for TCMR [restricted to Banff t ≥ 1 + i ≥ 1]) in indication biopsies.

 ○ However, in agreement with ESOT, the CHMP noted that there are clear between-center differences in performing protocol biopsies.
 ○ For regulatory purposes, the categorization of indication for renal transplant biopsy based on “per protocol” vs. “indication” may not be ideal.

• The CHMP commented that aTCMR (for both types of biopsies, protocol, and indication) and borderline changes (for indication biopsies only) could be primary efficacy endpoints for non-inferiority purposes, as the incidence of aTCMR is as low as 10%.

 ○ However, the concept of inferiority versus superiority is more applicable to the comparator type (approved vs standard of care) and not to the endpoint as such. Acceptance of a non-inferiority approach should be discussed *a priori.*

• The CHMP agreed that there is a need for more detailed analysis of the clinical relevance of minimal changes in the proposed histological subtypes of TCMR, including borderline changes in protocol biopsies.
